# Supplementary material for: Direct healthcare costs of lip, oral cavity and oropharyngeal cancer in Brazil
Source: PLoS One. 2021 Feb 17;16(2):e0246475. doi: 10.1371/journal.pone.0246475 (PMC7888595; doi:10.1371/journal.pone.0246475)
Supplement: Attachment — Submitted filename: Response to reviewer_2.pdf [file pone.0246475.s004.pdf]

January 6th, 2021

Dear Editor,

Thank you for considering a revised version of our manuscript **PONE-D-20-19121** entitled **'Direct healthcare costs of lip, oral cavity and oropharyngeal cancer in Brazil'**. All addressed points raised during the review process were considered and the manuscript changes are presented below. The manuscript line numbers cited on each answer below are in accordance with the file "manuscript_with_track_changes".

Yours sincerely,

The Authors

Reviewer 2, comment 1: "1. The issue (mentioned on line 278-281) of presentation of results in USD by other studies cannot be stated as a limitation of the present study. Firstly, it is not a limitation (of the methodology followed) of the present study. Secondly, authors can easily adjust the estimates from US$ to I$ based on the latest conversion and inflation rates. So, I think that the authors should try to report the results of the 'other small number of studies' (by adjusting the estimates to I$) on OC in the discussion section. "

Our response: Thank you for raising this question and for the opportunity to clarify and fix it. In fact, in the paragraph from lines 278-281 we intended to highlight limitations of our study but also, other aspects to be considered for further studies, which were not a limitation of our study. However, the text was not clear not only about that, but also regard the "small number of studies", which were already discussed on the first paragraph of the discussion and their results were reported on the second paragraph of discussion section (lines 213-224) adjusting their estimates to I$. The paragraph from lines 278-281 was rephrased (lines 296-299).

Reviewer 2, comment 2: The paragraphs are never three lines short. However, the paragraphs on lines 197-199, 259-161 and 262-266 are of just 3-4 lines. They can either be merged with the other paragraphs maintaining the flow of the discussion or can be deleted strategically without breaking the flow.

Our response: We agree with the suggestions and changes in the text were done aiming to maintain the flow of the discussion, which can be seen on lines: 199-212; 232-240; 296-299.

Reviewer 2, comment 3: The paragraph from lines 262-266 does not fit in the flow the discussion section. It could either be deleted or merged in the other paragraph on line 282-292. Similarly, the paragraph with lines 229-236, can be reduced (by summarizing the information presented in it) and merged with the subsequent paragraph.

Our response: Changes on paragraph from lines 262-266 can be seen on lines 271-276.The paragraph from lines 229-236 was merged with subsequent paragraph and summarized into the paragraph from lines 232-240.

Reviewer 2, comment 4: Information mentioned in the last 2 paragraphs (of the discussion section) specifically focuses on strategies to reduce the incidence of cancers. This information is quite important, but authors can try to summarize in a better and intelligible fashion in a short informative paragraph.

Our response: The last two paragraphs of discussion were summarized in a shorter paragraph from lines 300-309 and one reference (43*) was added to strength scientific support.

Reference 43*: Brocklehurst P, Kujan O, O'Malley LA, Ogden G, Shepherd S, Glenny AM. Screening programmes for the early detection and prevention of oral cancer. Cochrane Database Syst Rev. 2013;19(11):CD004150. doi: 10.1002/14651858.CD004150.
