# Supplementary material for: Direct healthcare costs of lip, oral cavity and oropharyngeal cancer in Brazil
Source: PLoS One. 2021 Feb 17;16(2):e0246475. doi: 10.1371/journal.pone.0246475 (PMC7888595; doi:10.1371/journal.pone.0246475)
Supplement: Attachment — Submitted filename: Reviewer comments_cost of oral cancer_Brazil.docx [file pone.0246475.s001.docx]

**Reviewer comments**

The study presents estimates of the direct medical expenditure incurred on oral cancer by the Brazilian health system. The study is really useful in defining the magnitude of the disease in terms of economic burden. Further, the estimates from the present study can also be used for assessing the efficiency of the present health system and strategies for reducing the health expenditure. Although, the study achieves its main objective of estimating the direct cost on OC, but I have a few concerns in the methodology followed:

1. The present analysis is actually a type of prevalence based cost of illness study. But, I am not confident (as per details mentioned in the methods section) that it uses the principles of top-down costing methods. The study simply measures the actual quantity of services delivered as the part of management of OC in Brazil (for the reference time period) and then multiply it with the cost of delivering these services. As the top down approach uses some type of aggregate data (morbidity, treatment utilization or total cost on health care delivery) which is then allocated to specific service or diseases. The authors should justify the use of top-down approach in the present study as the details of it in either data collection or data analysis is not clearly mentioned in the methods section.
2. The authors report that the data from Inpatient Information System (SIH-SUS) and the Outpatient Information System (SIA-SUS) was used to record hospitalization and outpatient information. It should be clearly mentioned that what type of information or data was assessed from the above mentioned resources. Whether only the utilization data on the number of hospitalizations, ICU admissions and outpatient procedures corresponding to the specific ICD-10 codes was extracted or the information on the specific resource use for each of the inpatient and outpatient procedure was also available from the data sources (as mentioned on the lines 114 -119 and 121-122). If the segregated information on resource use was not available (lines 119-120), how the inpatient costs were segregated into professional costs and hospital service costs/ICU costs (table 1).
3. Operational definition of professional cost (with regards to inpatient care) is missing (what cost components were included in this). ICU cost also seems to include some component of professional cost (cost of technical teams). Authors should take into consideration that is there is no duplication of costs.
4. The authors mentioned that expenses based on prices represented by the Brazilian Ministry of Health’s reimbursements to all health providers were used for estimating the cost. The manuscript lacks details on what type of information on prices was used from the reimbursement system.
   a. Whether, an estimate of average unit cost on outpatient procedures or inpatient stay/ICU stay was extracted from the reimbursement system and simply multiplied with the number of procedures to estimate the total direct cost.
   b. OR it is the actual amount reimbursed to the health care provider for each of the outpatient procedure or inpatient stay.
   c. OR prices of various resources consumed in the delivery of outpatient and inpatient care were extracted from the reimbursement system and then these were used to calculate the cost the treatment given.
5. Transport cost and cost of companion is usually included in the category of direct non-health care cost. Author can think of adjusting this issue.
6. Is there any other reason for the higher cost in oropharynx/floor of mouth/base of tongue besides the higher incidence of cancer for these specific sites? Or there was more utilization of outpatient procedures and inpatient services for the management of these specific cancer sites as compared to other sites. Similarly, what is the possible reason of ‘growing’ expenditure in the case of oropharynx as compared to stationary rates for other sites? It is important and should be mentioned in the discussion section.
7. Authors have mentioned total number of procedures and total cost of each procedure in table 2. It will be also useful to add cost per procedure in a separate column in the table.
8. It should be mentioned in table 1 that the cost are presented in I$ million dollars.
9. The authors mention that costs for skin cancer in Brazil can vary highly according to the stage of disease. If possible, the authors should provide the segregation of total direct cost on OC as per stage of diagnosis of the disease. This will further strengthen the argument.
10. The line 249-250 i.e., ‘presentation of the SIA-SUS and SIH-SUS values by number of procedures is an impairment to identifying the individual costs of OC”. Please try to rephrase it for better understanding.
11. Overall, the discussion section needs improvement in terms of clarity of the language for better understanding of the readers.
