# Supplementary material for: Direct healthcare costs of lip, oral cavity and oropharyngeal cancer in Brazil
Source: PLoS One. 2021 Feb 17;16(2):e0246475. doi: 10.1371/journal.pone.0246475 (PMC7888595; doi:10.1371/journal.pone.0246475)
Supplement: Attachment — Submitted filename: Reviewer comments_2.docx [file pone.0246475.s003.docx]

The authors have done a good work in terms of providing better explanation of the procedures/methodology followed and a background on the health information system in the methods section of the manuscript. But, I feel that the discussion can still be improved in terms of better structuring, content and flow. Below are few suggestions:

1. The issue (mentioned on line 278-281) of presentation of results in USD by other studies cannot be stated as a limitation of the present study. Firstly, it is not a limitation (of the methodology followed) of the present study. Secondly, authors can easily adjust the estimates from US$ to I$ based on the latest conversion and inflation rates. So, I think that the authors should try to report the results of the ‘other small number of studies’ (by adjusting the estimates to I$) on OC in the discussion section.
2. The paragraphs are never three lines short. However, the paragraphs on lines 197-199, 259-161 and 262-266 are of just 3-4 lines. They can either be merged with the other paragraphs maintaining the flow of the discussion or can be deleted strategically without breaking the flow.
3. The paragraph from lines 262-266 does not fit in the flow the discussion section. It could either be deleted or merged in the other paragraph on line 282-292. Similarly, the paragraph with lines 229-236, can be reduced (by summarizing the information presented in it) and merged with the subsequent paragraph.
4. Information mentioned in the last 2 paragraphs (of the discussion section) specifically focuses on strategies to reduce the incidence of cancers. This information is quite important, but authors can try to summarize in a better and intelligible fashion in a short informative paragraph.
